# Older Americans Act Supports and Services: Adapting to the Pandemic

**DOI:** 10.1093/geroni/igab046.1165

**Published:** 2021-12-17

**Authors:** Amy Gotwals

**Affiliations:** USAging, Washington, District of Columbia, United States

## Abstract

This session provides insights into how the pandemic challenged the capabilities and ingenuity of the Older Americans Act (OAA) programs and the aging network. Speakers will include key aging network stakeholders, who will discuss the overnight evolution of programs serving often isolated older adults.

